# Human resources for eye health: ensuring a smooth pipeline

**Published:** 2018-07-31

**Authors:** Daksha Patel

**Affiliations:** 1 E-learning Director: International Centre for Eye Health, London School of Hygiene and Tropical Medicine, London, UK.

**Health workforce planning improves when activities in the education system align with labour market dynamics.**

**Figure:**
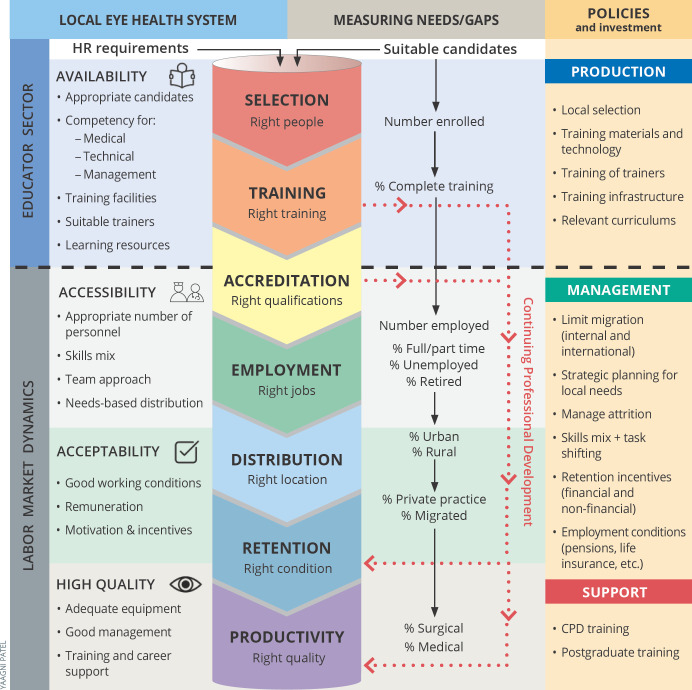


The health workforce ‘pipeline’^1^ depicted in the infographic above shows what is required to produce a functional eye health workforce.

It suggests ways to measure gaps and progress, and highlights the essential role of education and continuing professional development to support service provision.

The four E's (**E**ntry planning, **E**ntry, **E**xist, **E**xit) highlight the four stages in the life cycle of the health workforce – each of which requires careful planning and adequate investment.

- **Entry planning.** Understanding the need for eye health personnel and working out how many candidates to recruit, as some are likely to fail or drop out
- **Entry.** Training the workforce
- **Exist.** Managing the workforce and their working conditions
- **Exit.** Keeping track of the number of workers who retire, start to work part time or migrate.

Investment to improve retention and limit attrition (loss of staff members due to reasons other than death or retirement) includes establishing functional working conditions along with realistic financial and non-financial incentives.

Universal health coverage^2^ relies on the availability of the workforce and their accessibility as a result of equitable distribution, particularly in rural areas.

Universal health coverage also depends on the quality of their performance – initially achieved through mastery of appropriate competencies and maintained after graduation through ongoing continuing professional development. It is essential that health workers are fit for practice – and remain so.
